# Tissue-Level Heterogeneity in Raw Giant Juncao Straw and Its Influence on Ammonium and Phosphate Adsorption

**DOI:** 10.3390/polym18202594

**Published:** 2026-10-21

**Authors:** Xiaomei Wu, Chaohui Chen, Wenting Ren, Rilong Yang, Junyue Shan, Yan Yu

**Affiliations:** 1 College of Materials Engineering, Fujian Agriculture and Forestry University, Fuzhou 350108, China; xiaomeizi163@126.com (X.W.);; 2 Institute of Food Science and Technology, Fujian Academy of Agricultural Sciences, Fuzhou 350003, China; 3 Key Laboratory of Plant Fiber Functional Materials, National Forestry and Grassland Administration, Fuzhou 350002, China

**Keywords:** Giant Juncao, lignocellulosic biomass, tissue heterogeneity, ammonium, phosphate, adsorption

## Abstract

Raw lignocellulosic biomass offers a low-input route for nutrient capture, yet its internal tissue heterogeneity is rarely considered when adsorption behavior is interpreted. Here, Giant Juncao (*Pennisetum giganteum*; also reported as *Cenchrus fungigraminus*) straw was separated into rind and pith to determine how native tissue architecture and cell-wall chemistry relate to ammonium (NH_4_^+^) and phosphate adsorption. The pith occupied 72.33% of stem volume but only 26.85% of dry mass and had a much lower basic density than the rind (0.072 vs. 0.570 g·cm^−3^). It also showed a larger N_2_-accessible specific surface area (2.67 vs. 1.13 m^2^·g^−1^), greater pore volume (0.006 vs. 0.001 cm^3^·g^−1^), higher pectin content (20.58 vs. 14.81 mg·g^−1^), and lower cellulose crystallinity (48.28% vs. 57.30%). These contrasts were accompanied by tissue-dependent NH_4_^+^ uptake: the rind and pith adsorbed 4.19 ± 1.02 and 10.36 ± 1.54 mg·g^−1^, respectively, whereas whole straw reached 13.80 ± 0.23 mg·g^−1^ under the same comparison conditions. Phosphate uptake was less tissue-sensitive (19.57–22.40 mg·g^−1^). More than 90% of the 24 h uptake was reached within 60 min. Nonlinear least-squares fitting, evaluated by multiple criteria (R^2^, SSE, RMSE, χ^2^, or AIC), identified the pseudo-second-order kinetic model and the Langmuir–Freundlich isotherm as the best empirical descriptions of the measured data. Spectroscopic changes were consistent with participation of oxygen-containing cell-wall groups in nutrient retention, but did not uniquely resolve molecular binding pathways. The results show that tissue-scale architecture and heterogeneous lignocellulosic chemistry jointly shape nutrient uptake in minimally processed Giant Juncao and support further evaluation of the unseparated feedstock in real wastewater and nutrient-reuse scenarios.

## 1. Introduction

Agricultural and forestry biomass is generated on a very large scale and is increasingly considered a renewable feedstock for materials, energy, and environmental applications [1,2]. For sorption-based water treatment, minimally processed lignocellulosic biomass is particularly attractive because it avoids the energy demand and chemical inputs associated with carbonization, activation, or extensive surface modification. Its adsorption performance, however, is commonly interpreted from the composition of bulk powders, even though plant stems are intrinsically heterogeneous biological materials.

Giant Juncao (*Pennisetum giganteum* Z.X. Lin; also reported as *Cenchrus fungigraminus*) is a high-biomass C4 grass used for forage, mushroom cultivation, bioenergy, and lignocellulosic products [3,4,5,6,7]. Its stem architecture has also been exploited in water-management and solar-interfacial-evaporation materials [8]. Macroscopically, the stem comprises a dense outer rind surrounding a lightweight pith. Similar rind–pith differentiation occurs in sunflower stalk, Miscanthus, corn stover, and other grass-like biomasses, where tissue identity strongly influences chemical composition, fractionation behavior, and material processing [9,10,11,12].

At the cellular level, Giant Juncao contains parenchyma cells, fibers, vessels, sieve elements, and vascular-bundle-associated tissues with markedly different wall thicknesses, lumen dimensions, densities, and degrees of lignification. Previous studies have characterized fiber morphology and bulk lignocellulosic composition of Giant Juncao [13,14], but averaged whole-stem values can obscure the structural and chemical contrast between rind and pith. This distinction is potentially important for adsorption because solute transport in raw biomass occurs through a hierarchy of macroscopic lumina, vascular pathways, fractured cell walls, and chemically distinct polymer-rich surfaces.

Nitrogen- and phosphorus-rich livestock wastewaters contribute to eutrophication when inadequately managed [15,16,17]. Adsorption can provide a relatively simple route for decentralized nutrient capture and, when biomass sorbents are used, may enable recovery of nutrient-containing solids [18,19]. Many high-capacity sorbents nevertheless rely on pyrolysis, chemical activation, or loading of Mg-, Ca-, Fe-, or other mineral phases [20,21,22]. Raw lignocellulosic feedstocks are unlikely to reach the capacities of such engineered materials, but they may offer value where low processing intensity, local availability, and direct reuse are more important than record adsorption capacity.

Raw agricultural straws consist primarily of cellulose, hemicelluloses, lignin, pectic polysaccharides, extractives, and endogenous inorganic constituents [23]. Their nutrient uptake may therefore reflect a combination of accessible cellular pathways, ionizable oxygen-containing groups, ion exchange, hydrogen-bonding or other polar interactions, and mineral-mediated association [24,25]. Because these features are distributed unevenly among plant tissues, treating the stem as a uniform adsorbent can mask structure–function relationships that are both scientifically and practically relevant.

Accordingly, this study tested the hypothesis that nutrient adsorption by raw Giant Juncao emerges from the combined effects of tissue-scale architecture and chemically heterogeneous cell-wall domains. We first compared the anatomy, physical properties, and lignocellulosic chemistry of isolated rind and pith, and then compared their NH_4_^+^ and PO_4_^3−^ uptake with that of unseparated whole straw in single- and binary-solute systems. Whole-straw adsorption was further evaluated as a function of contact time, initial concentration, adsorbent dosage, pH, and coexisting ions, followed by kinetic and equilibrium modeling. FTIR, XRD, and XPS were used to identify adsorption-associated spectral changes. The objective was not to assign a unique molecular binding mechanism from indirect characterization, but to establish a defensible tissue-structure–adsorption relationship for minimally processed biomass.

## 2. Materials and Methods

### 2.1. Giant Juncao Collection and Sample Preparation

**Figure 1:**
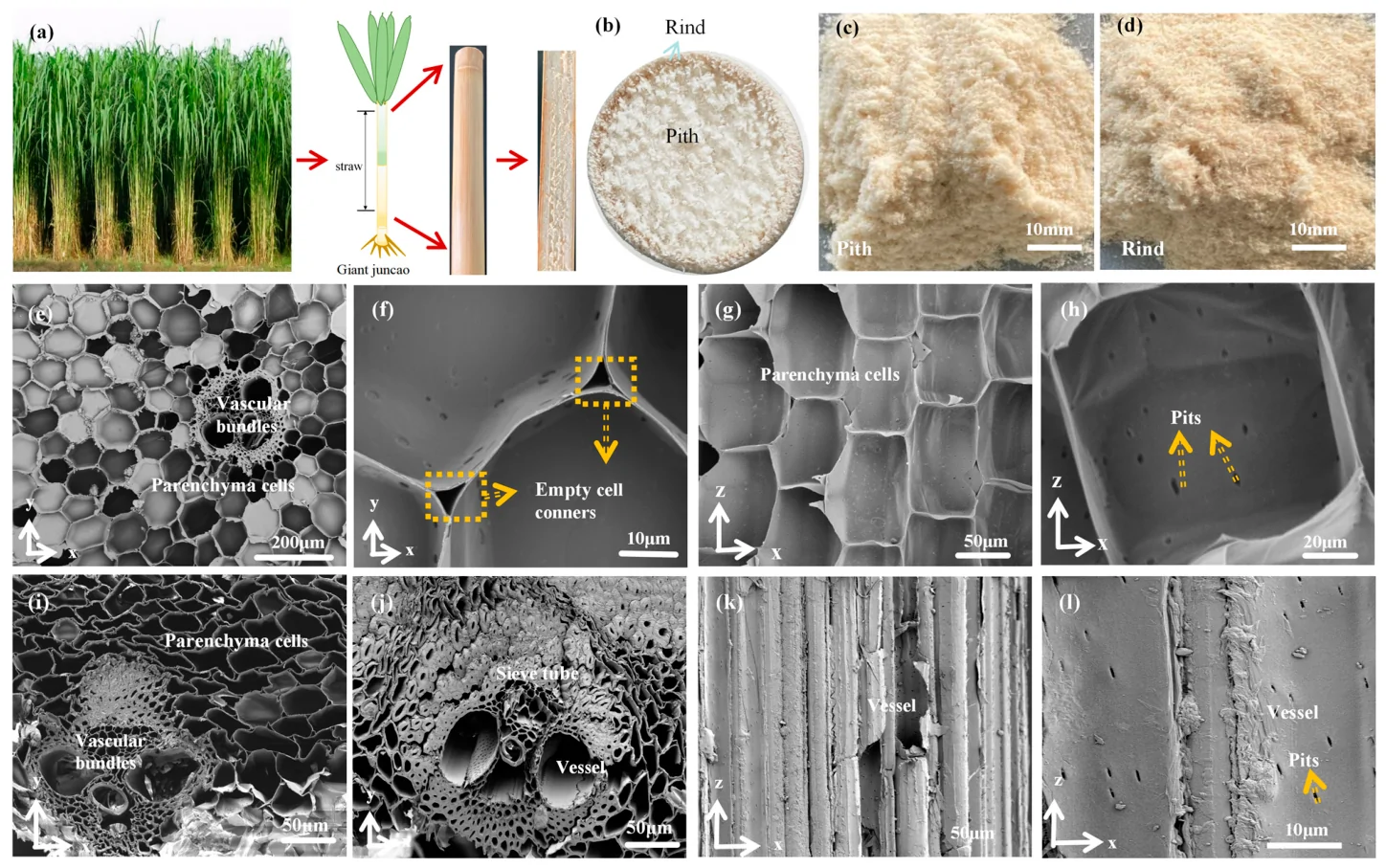
Hierarchical rind–pith heterogeneity of Giant Juncao straw. (**a**) Plant and stem sampling scheme; (**b**) representative stem cross-section identifying rind and pith; (**c**,**d**) macroscopic appearance of separated pith and rind, respectively; (**e**,**f**) transverse pith microstructure; (**g**,**h**) longitudinal pith microstructure showing parenchyma, lumina, and pits; (**i**,**j**) transverse rind microstructure showing the denser vascular/fibrous organization; (**k**,**l**) longitudinal rind microstructure showing axial vessels and pits.

Giant Juncao straw was collected in November 2023 from the JUNCAO Plantation Base at the University Town Campus of Fujian Agriculture and Forestry University, Fuzhou, China. Stalks were harvested approximately 20 cm above the root base (Figure 1a). Leaf sheaths were removed, and the stalks were washed, cut into 5–8 cm segments, and air-dried. For tissue-specific experiments, the dried stalks were manually separated with a blade into the outer rind and inner pith (Figure 1b–d). The two fractions were ground separately under identical conditions and sieved to 30–60 mesh (250–600 μm), minimizing particle-size differences in the rind–pith comparison. The sieved samples were dried at 105 °C to constant mass and stored in sealed containers before characterization and adsorption tests. Whole-straw powder was prepared without rind–pith separation for comparison and for subsequent parametric adsorption experiments.

### 2.2. Characterization

For basic density and tissue fractions, approximately 3 cm stem segments were weighed and measured before and after oven drying. The rind and pith were separated for determination of their dry-mass fractions, while cross-sectional dimensions were obtained from digital images with ImageJ 1.54p (National Institutes of Health, Bethesda, MD, USA) analysis. Basic density was calculated from oven-dry mass and measured volume. Six replicate stem segments were used for these measurements.

Microstructure was observed by Field-Emission Scanning Electron Microscopy (FE-SEM; FEI Nova NanoSEM 230, Hillsboro, OR, USA) at an accelerating voltage of 5 kV and a working distance of 5 mm after platinum sputter-coating. Transverse and longitudinal sections were used to identify vascular bundles, parenchyma cells, lumina, and pits. N_2_ adsorption–desorption measurements were performed at −196.15 °C using an ASAP 2460 analyzer (Micromeritics, Norcross, GA, USA) after vacuum degassing at 80 °C for 10 h. BET specific surface area and pore volume were used for comparative assessment of N_2_-accessible porosity. Zeta potential was measured using a Malvern Zetasizer Nano ZS90. Surface functional groups were characterized by attenuated-total-reflectance Fourier-transform infrared (ATR-FTIR) spectroscopy (Spotlight 400, PerkinElmer, Waltham, MA, USA) over 400–4000 cm^−1^ at 4 cm^−1^ resolution. Thermogravimetric analysis was performed using an SDT Q600 analyzer (TA Instruments, New Castle, DE, USA) at 10 °C min^−1^ under nitrogen. Dynamic water-vapor sorption was measured using a DVS Resolution analyzer (Surface Measurement Systems, London, UK). X-ray diffraction (XRD; Ultima IV, Rigaku, Japan) was used to characterize cellulose crystalline structure; relative crystallinity was calculated using the peak-height approach employed in the source dataset. X-ray photoelectron spectroscopy (XPS; K-Alpha, Thermo Fisher Scientific, Waltham, MA, USA) was used to compare surface chemical states before and after nutrient adsorption.

The C contents of the samples were determined using a carbon/sulfur analyzer (CS844, LECO Corporation, St. Joseph, MI, USA), while the O, N, and H contents were analyzed with an oxygen/nitrogen/hydrogen analyzer (EMGA-830, HORIBA, Ltd., Kyoto, Japan). Cellulose, hemicellulose, lignin, extractives, and ash were determined using the analytical procedures of NREL-based compositional analysis. Monosaccharides were determined by ion chromatography (Dionex ICS-5000+, Thermo Scientific), and pectin was measured according to GB 10742-08. Composition measurements were performed in triplicate. Gel-state two-dimensional heteronuclear single-quantum coherence nuclear magnetic resonance (2D-HSQC NMR) followed Zhang et al. [26]. Briefly, ball-milled rind and pith powders (20 mg; 500 rpm for 5 h) were dispersed in 0.5 mL DMSO-d_6_ and sonicated to form homogeneous gels. Spectra were acquired on a Bruker AVIII 800 MHz NMR spectrometer equipped with a cryogenic probe (Bruker BioSpin, Rheinstetten, Germany). The HSQC-derived linkage and aromatic-unit data were treated semiquantitatively.

### 2.3. Adsorption Experiments

Ammonium chloride (NH_4_Cl) and potassium dihydrogen phosphate (KH_2_PO_4_) were dissolved in distilled water to prepare ammonium and phosphate solutions. All ammonium concentrations and adsorption capacities are expressed as NH_4_^+^ (mg·L^−1^ and mg·g^−1^) and phosphate concentrations and adsorption capacities are expressed as PO_4_^3−^ (mg·L^−1^ and mg·g^−1^). Unless otherwise stated, batch adsorption was conducted in 250 mL Erlenmeyer flasks containing 100 mL of solution at 25 °C and 150 rpm. After the prescribed contact time, suspensions were filtered through 0.45 μm aqueous membrane filters. Residual NH_4_^+^ was determined by the Nessler method at 420 nm, and PO_4_^3−^ was measured by the molybdenum-blue method at 700 nm using a Lambda 750 UV–visible spectrophotometer. Blank and leaching controls were performed for both NH_4_^+^ and PO_4_^3−^. For the blank controls, the target solutions without adsorbent were shaken under conditions identical to the adsorption experiments. For the leaching controls, the adsorbents were shaken in solute-free background electrolyte under the same conditions, and the released native NH_4_^+^ and PO_4_^3−^ were quantified (Figure S3). All adsorption capacities reported in this study are net values, corrected by subtracting both the blank loss and the leached background from the corresponding equilibrium concentrations.

The equilibrium adsorption capacity, q_e_ (mg·g^−1^), was calculated as q_e_ = (C_0_ − C_e_)V/m, where C_0_ and C_e_ are the initial and equilibrium aqueous concentrations (mg·L^−1^), V is the solution volume (L), and m is the dry adsorbent mass (g). Removal efficiency was calculated as (C_0_ − C_e_)/C_0_ × 100%.

The effects of adsorbent dosage, initial pH, contact time, and initial solute concentration were investigated using a single-factor design: in each experiment, one parameter was varied over the indicated range while all other parameters were held constant at the reference conditions. Unless otherwise specified, the reference conditions for all batch experiments were: initial NH_4_^+^ and PO_4_^3−^ concentrations of 100 mg·L^−1^, adsorbent dosage of 1.0 g·L^−1^ (0.1 g per 100 mL), and initial pH of 6.0. Adsorbent dosage ranged from 0.25 to 10 g·L^−1^ (0.25, 0.5, 1.0, 2.5, 5.0, 7.5, and 10 g·L^−1^); initial NH_4_^+^ and PO_4_^3−^ concentrations were 10, 20, 30, 40, 50, 100, 200, and 300 mg·L^−1^; initial pH ranged from 3 to 10 (adjusted with 1 mol·L^−1^ NaOH or HCl); and contact time was extended to 24 h. To quantify the contribution of NH_3_ volatilization, blank experiments without adsorbent were performed under conditions identical to those of the adsorption experiments (initial NH_4_^+^ concentration of 100 mg·L^−1^, the same pH values, contact time of 24 h, 25 °C, and 150 rpm) at each pH level, and the apparent NH_4_^+^ adsorption capacities were corrected accordingly. The effects of coexisting cations (Na^+^, K^+^, Cu^2+^, Zn^2+^, Ca^2+^, Mg^2+^, and Fe^3+^) on NH_4_^+^ uptake and coexisting anions (NO_3_^−^, Cl^−^, HCO_3_^−^, CO_3_^2−^, and SO_4_^2−^) on PO_4_^3−^ uptake were evaluated at competing-ion concentrations of 20–100 mg·L^−1^. Because phosphate speciation changes with pH, the term “phosphate” is used throughout rather than implying that PO_4_^3−^ is the dominant aqueous species under all test conditions. For the competing-ion experiments, the pH of each solution was measured and re-adjusted to 6.0 immediately after the addition of the competing ion, so that all systems shared the same initial pH as the single-ion adsorption experiments. No precipitate or turbidity was observed in any of the competing-ion systems during the 24 h contact period. Each treatment was conducted in triplicate.

Kinetic data were fitted using pseudo-first-order (PFO), pseudo-second-order (PSO), and Elovich models. Equilibrium data were fitted with Langmuir, Freundlich, and Langmuir–Freundlich equations. The model forms and fitting parameters are provided in Supplementary Section S1 and Table S6–S10. These models were used as empirical descriptors; goodness of fit was not treated as independent proof of a specific molecular adsorption mechanism.

### 2.4. Statistical Analysis

All adsorption experiments were performed in triplicate (*n* = 3, technical replicates), and the results are expressed as mean ± standard deviation. One-way analysis of variance (ANOVA) followed by Tukey’s HSD post hoc test (*p* < 0.05) was conducted to evaluate the statistical significance. Different lowercase letters in the figures indicate statistically significant differences. All statistical analyses were performed using Origin 2023.

## 3. Results and Discussion

### 3.1. Micromorphology and Physical Properties

The Giant Juncao stem showed a pronounced anatomical separation between a dense outer rind and a voluminous inner pith (Figure 1a–d). SEM revealed that the pith was dominated by thin-walled parenchyma interspersed with vascular bundles (Figure 1e–h). Parenchyma lumina were approximately 60–88 μm in diameter, whereas the reported wall thicknesses were 0.24–0.45 μm. By contrast, the rind contained a denser arrangement of vascular and supporting tissues (Figure 1i–l), with smaller parenchyma lumina (17.12–36.91 μm) and much greater reported wall thicknesses (6.27–9.29 μm). Longitudinal views showed axial conduits and pits in both tissues. These micrometer-scale pathways are substantially larger than the pores probed by N_2_ sorption and are therefore important when considering liquid-phase access in raw plant tissue [27].

The physical measurements quantified this anatomical contrast. The rind had a basic density of 0.570 ± 0.011 g·cm^−3^, almost eight times that of the pith (0.072 ± 0.0068 g·cm^−3^). The pith accounted for 72.33% of stem volume but only 26.85% of dry mass, whereas the rind represented 27.67% of volume and 73.15% of dry mass. The measured moisture contents were 45.15 ± 4.19% for the rind and 41.23 ± 4.21% for the pith (Table S1). Thus, on a mass basis, pith corresponds to a much larger cellular volume than rind. For adsorption, this distinction matters because solute access depends not only on nanometer-scale surface area but also on lumina, fractured parenchyma, vascular channels, and the distance to chemically active cell-wall surfaces. Similar functional partitioning between dense outer tissues and low-density pith has been reported for other stalk biomasses [9,12].

**Figure 2:**
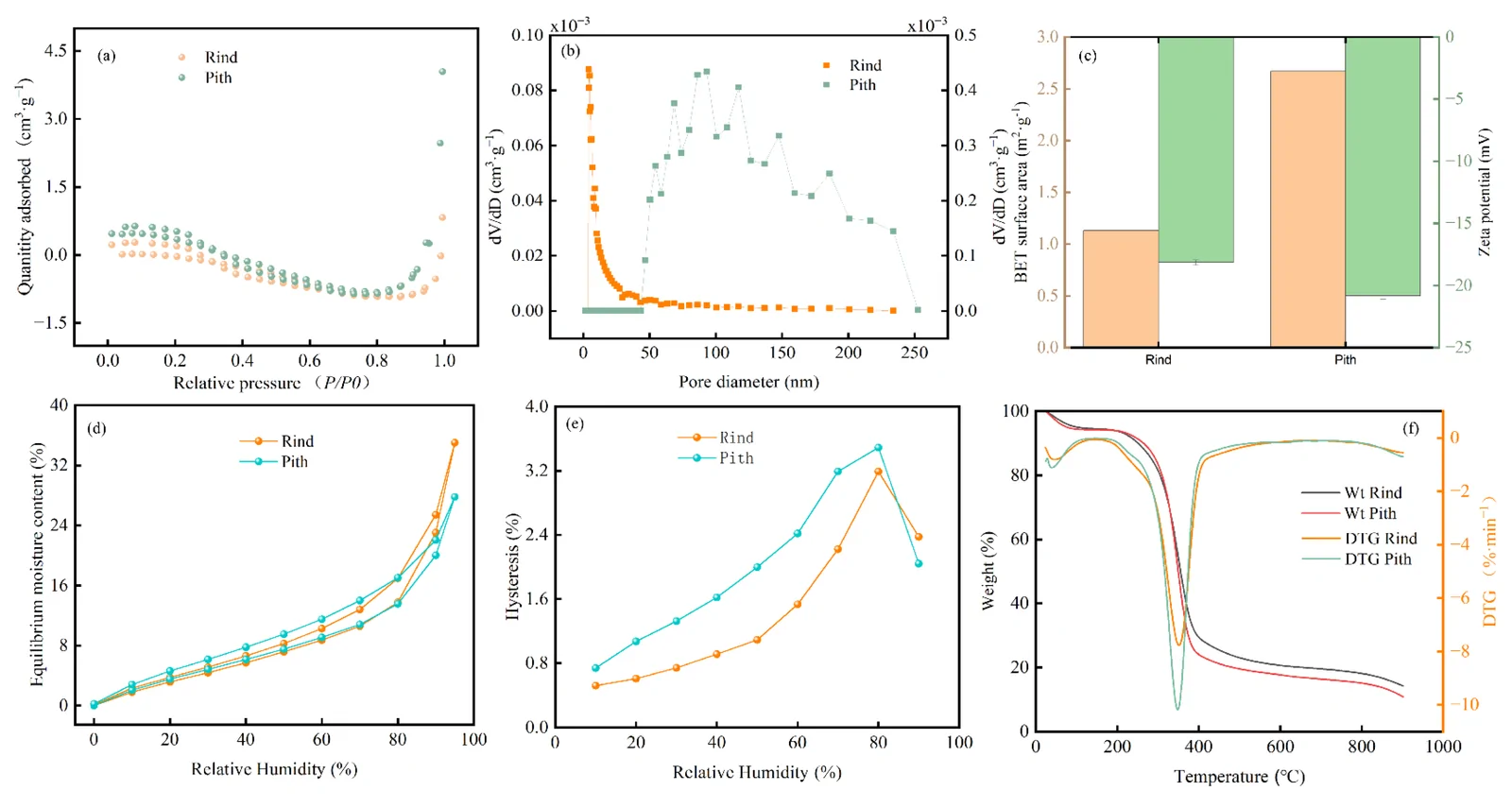
Contrasting physical properties of Giant Juncao rind and pith. (**a**) N_2_ adsorption–desorption isotherms; (**b**) reported pore-size distributions; (**c**) BET specific surface area and zeta potential; (**d**) dynamic water-vapor sorption–desorption isotherms; (**e**) moisture-sorption hysteresis; (**f**) TG and DTG curves. In all panels, orange represents the rind and green represents the pith.

N_2_ sorption gave low absolute BET areas for both raw tissues, as expected for unactivated lignocellulosic biomass (2.67 and 1.13 m^2^·g^−1^ for pith and rind, respectively; pore volume 0.006 and 0.001 cm^3^·g^−1^) (Figure 2a–c). We note that these N_2_-accessible areas are far too small, in absolute terms, to accommodate the measured ammonium uptakes, indicating that adsorption from aqueous solution occurs predominantly at internal lumen walls and hydrated cell-wall interfaces that N_2_ cannot probe. The roughly twofold difference in BET area between the two tissues is therefore used only as a directional, comparative indicator of relative porosity, and is not invoked as an explanation of the difference in uptake; the structurally decisive feature of the pith is instead its much larger lumen-scale volume revealed by SEM (Figure 2d–f), together with its greater pectin content. These values should be interpreted comparatively rather than as evidence of a highly developed microporous adsorbent. They complement, but do not replace, the much larger lumen-scale differences observed by SEM. Zeta potentials were negative for both tissues (−18.13 ± 0.22 mV for rind and −20.83 ± 0.26 mV for pith), which is directionally favorable for cation association but argues against explaining phosphate uptake by simple electrostatic attraction to a uniformly negative surface.

The dynamic vapor-sorption curves (Figure 2d,e) were sigmoidal for both tissues. Below approximately 80% relative humidity (RH), the pith showed slightly greater equilibrium moisture uptake, whereas at 95% RH the rind reached a higher equilibrium moisture content (35.02% vs. 27.80%). Both tissues displayed sorption hysteresis, with the largest differences occurring at intermediate RH (3.19% for rind and 3.49% for pith). These trends likely reflect combined effects of cell-wall composition, accessible sorption sites, and tissue architecture. They indicate that the two tissues establish different hydrated interfacial environments, which may influence aqueous transport and ion access during adsorption.

TG and DTG results are summarized in Figure 2f and Tables S2 and S3. Both tissues showed an initial drying region, a low-mass-loss transition region, a major devolatilization region, and a high-temperature residual-decomposition region. The main mass loss was 66.62% for rind and 70.84% for pith. The lower comprehensive pyrolysis index, D, of the rind indicates lower overall pyrolysis reactivity under the tested heating conditions [28]. These thermal differences are consistent with the much greater lignification of the rind and the more weakly lignified parenchymatous character of the pith.

### 3.2. Chemical Composition of Rind and Pith

**Figure 3:**
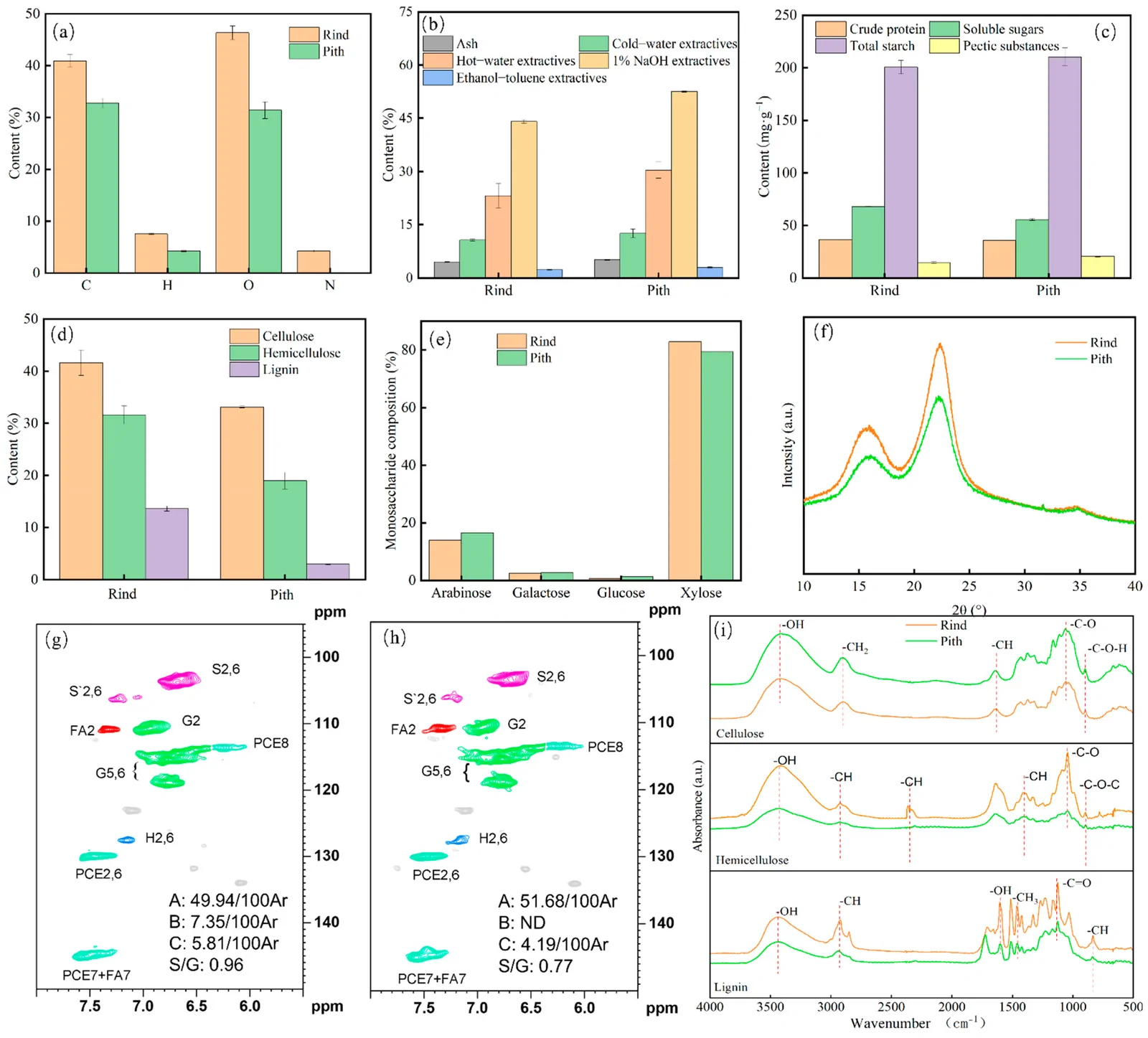
Chemical composition of Giant Juncao rind and pith. (**a**) Bulk elemental composition; (**b**) extractive and ash contents; (**c**) crude protein, soluble sugars, total starch, and pectic substances; (**d**) cellulose, hemicellulose, and lignin contents; (**e**) monosaccharide composition; (**f**) XRD patterns with relative crystallinity values; (**g**,**h**) aromatic regions of 2D-HSQC NMR spectra of rind and pith, respectively; (**i**) FTIR spectra of isolated cellulose, hemicellulose, and lignin fractions.

Bulk elemental compositions are shown in Figure 3a. The rind contained more C, H, and N than the pith (for example, C: 40.90 ± 1.21% vs. 32.78 ± 0.89%; N: 4.25 ± 0.20% vs. 0.073 ± 0.01%). Because CHNO analysis averages all constituents within each tissue, these bulk values were treated as compositional descriptors rather than direct measures of surface binding-site density. In particular, mechanistic conclusions about adsorption were based on the combined structural and spectroscopic evidence rather than on elemental ratios alone.

The pith contained more cold-water, hot-water, and 1% NaOH extractives and more ash than the rind, whereas the ethanol–toluene extractives were similar (Figure 3b). Water-soluble fractions can include low-molecular-weight sugars, pectic substances, and inorganic salts, while dilute alkali also extracts portions of hemicellulose and low-molecular-weight lignin [29]. The resulting contrast is consistent with the different biological functions of the two tissues: the rind is specialized for mechanical support and protection, whereas the pith is dominated by storage-type parenchyma and less extensively lignified cell walls.

Major lignocellulosic differences were also evident (Figure 3d). The rind contained 41.62 ± 0.02% cellulose, 31.62 ± 0.77% hemicellulose, and 13.62 ± 0.0046% lignin, whereas the pith contained 33.13 ± 0.0018%, 18.98 ± 0.61%, and 2.94 ± 0.0010%, respectively. The low lignin content of the pith is consistent with its thin-walled parenchymatous structure. The pith was richer in pectic substances than the rind (20.58 vs. 14.81 mg g^−1^; Figure 3c). Pectic polysaccharides provide carboxyl and hydroxyl groups and form highly hydrated cell-wall domains; deprotonated carboxyl groups can contribute to cation retention. By contrast, negatively charged carboxylate sites do not provide a simple electrostatic explanation for phosphate uptake, for which polar interactions, protonated sites, and endogenous-mineral-mediated association may contribute. Both tissues retained the cellulose I diffraction pattern, but the relative crystallinity was higher in rind (57.30%) than in pith (48.28%) (Figure 3f). Detailed XRD peak fitting is provided in Figure S1 and Table S4.

Gel-state whole-cell-wall 2D-HSQC NMR was used for semiquantitative comparison of lignin interunit-linkage and aromatic correlations without prior lignin isolation [26]. The side-chain regions are shown in Figure S2, while aromatic regions are included in Figure 3g,h; the derived values are summarized in Table S5. β-O-4′ correlations were dominant in both tissues: 49.94 per 100 aromatic units in rind and 51.68 per 100 aromatic units in pith, corresponding to 79.15% and 92.49%, respectively, of the quantified A–C side-chain linkages. β-β′ correlations accounted for 11.65% of the quantified linkages in rind but were not detected in pith under the present measurement conditions; β-5′ correlations accounted for 9.20% and 7.51%, respectively. These data indicate a more condensed lignin linkage distribution in the rind and a more β-O-4′-rich distribution in the pith. Signals consistent with γ-acylated units, including p-coumarate-associated correlations, were observed in both tissues. Although differences in acylation and monolignol coupling may contribute to the linkage contrast, the HSQC data alone do not establish a unique biosynthetic cause for the non-detected β-β′ signal in pith [30].

The aromatic region indicated G-dominated H/G/S lignins in both tissues (S/G < 1): G 48.54%, S 46.46%, and H 5.00% for rind (S/G = 0.96), compared with G 53.21%, S 40.81%, and H 5.98% for pith (S/G = 0.77) (Table S5). p-Coumarate ester (PCE) and ferulate (FA) correlations were detected in both samples, consistent with the hydroxycinnamate chemistry of grass cell walls. An S′_2,6_ correlation near δC/δH 106.4/7.29 was also observed and assigned to oxidized syringyl-type structures. Collectively, these spectra show that rind–pith heterogeneity extends beyond total lignin content to lignin linkage and aromatic-unit distributions.

Taken together, the chemical analyses show that the rind and pith are not simply density variants of the same material. The rind is more highly lignified and has a more condensed lignin linkage distribution, whereas the pith is less lignified, richer in pectic substances, and dominated by β-O-4′ correlations. These distinctions provide a chemically plausible basis for different hydrated interfaces and ion-accessible environments, while not by themselves proving which individual polymer domain controls adsorption.

FTIR spectra of the isolated cellulose, hemicellulose, and lignin fractions (Figure 3i) showed the expected O–H, C–H, C–O–C, glycosidic, and aromatic skeletal bands of lignocellulosic polymers. Differences in band shape and relative intensity were observed between tissues, but these spectra were interpreted qualitatively because intensity differences can also arise from sample preparation and normalization. The key implication for adsorption is therefore the presence of multiple oxygen-containing polymer environments rather than a claim that a single FTIR band identifies a unique binding site.

### 3.3. Rind and Pith Contribute Differently to Nutrient Adsorption

**Figure 4:**
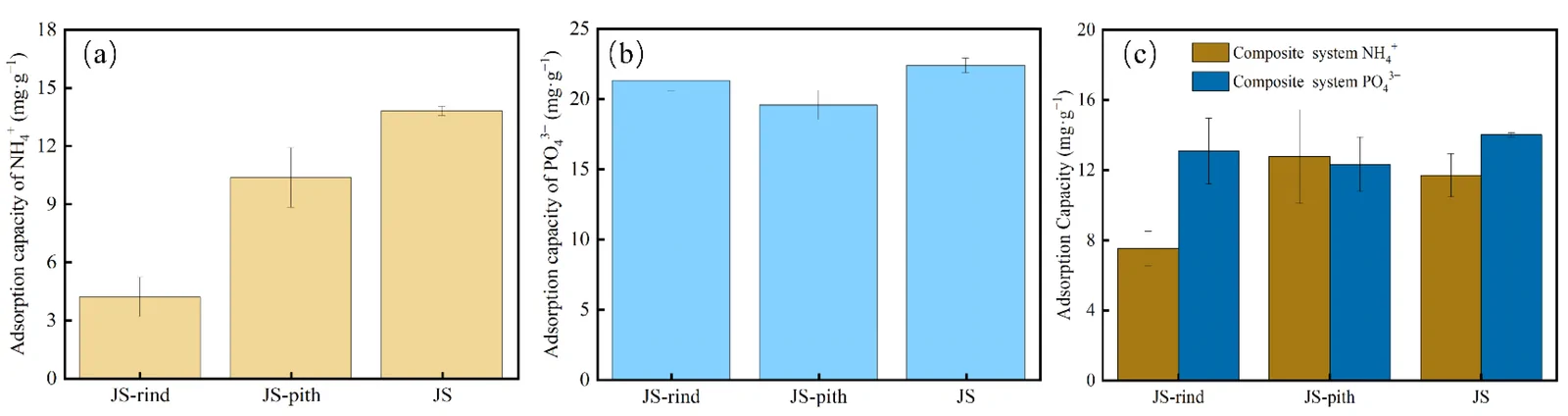
Tissue-dependent nutrient adsorption by Giant Juncao straw. (**a**) Equilibrium NH_4_^+^ adsorption capacities of isolated rind, isolated pith, and whole straw in the single-solute system; (**b**) corresponding PO_4_^3−^ capacities; (**c**) NH_4_^+^ and PO_4_^3−^ capacities of rind, pith, and whole straw in the binary-solute system. Different lowercase letters indicate statistically significant differences among adsorbents (one-way ANOVA with Tukey’s HSD test, *p* < 0.05).

The tissue comparison provided a functional test of the structural characterization. In the single-solute ammonium system, equilibrium uptake increased from 4.19 ± 1.02 mg·g^−1^ for the rind to 10.36 ± 1.54 mg·g^−1^ for the pith (Figure 4a), consistent with the pith’s larger accessible cellular volume, more negative zeta potential, and higher pectin content. Since these properties co-vary between the two tissues, their individual contributions cannot be isolated; the higher pectin content is considered the most plausible contributor, as pectin carboxyl groups are well-documented ammonium-binding sites [31].

Whole-straw NH_4_^+^ uptake reached 13.80 ± 0.23 mg·g^−1^, exceeding both isolated fractions, whereas the mass-weighted estimate predicted only ~5.85 mg·g^−1^ if the rind and pith behaved independently. To examine this non-additivity, the crushed rind and pith were re-mixed at their original mass ratio (0.7315:0.2685) and tested in parallel; the mixture’s capacity (10.93 mg·g^−1^ Figure S4) far exceeded the weighted value but remained below that of the intact straw. This confirms that single-point mass weighting is not a rigorous baseline, as adsorption capacity varies nonlinearly with equilibrium concentration, and that part of the accessible surface of the intact straw is lost during separation and drying. Indeed, the BET area of the intact straw (3.07 m^2^·g^−1^) was nearly twice the mass-weighted value of the fractions (1.54 m^2^·g^−1^), and NH_4_^+^ uptake was nearly proportional to BET area (3.7–4.5 mg·m^−2^) within the present samples, whose surface chemistries are broadly similar. This proportionality does not imply that physical surface area determines the absolute adsorption capacity—governed mainly by surface chemical sites (e.g., carboxyl groups and mineral-associated cations)—but rather explains why the intact straw retains a higher capacity than the mass-weighted sum of its parts. Distinguishing the underlying causes of this non-additivity (e.g., altered surfaces, soluble components, or interfacial environments) would require further tests on mixtures spanning a range of rind:pith ratios.

Phosphate showed a different tissue dependence (Figure 4b,c). The rind, pith, and whole straw adsorbed 19.57 ± 1.04, 22.40 ± 0.52, and 21.32 ± 0.72 mg·g^−1^, respectively. The rind–pith difference was modest, and the whole-straw value was close to the mass-weighted expectation (approximately 20.33 mg·g^−1^). Thus, the much larger pith lumen volume and BET area did not translate into a proportionally larger phosphate capacity. This behavior is consistent with phosphate retention depending more strongly on the chemistry of accessible sites and endogenous minerals than on geometric accessibility alone. Because both tissues had negative zeta potentials, nonspecific electrostatic attraction to a uniformly negative surface is unlikely to dominate phosphate uptake.

In the binary-solute system (Figure 4c), the PO_4_^3−^ uptake of all samples decreased significantly relative to the single-solute condition (e.g., 21.32 ± 0.72 → 13.08 ± 1.88 mg·g^−1^ for the rind and 22.40 ± 0.52 → 14.00 ± 0.49 mg·g^−1^ for the whole straw; *p* < 0.01). In contrast, the NH_4_^+^ response was matrix-dependent: the uptake by the isolated rind increased significantly (4.19 ± 1.02 → 7.53 ± 0.98 mg·g^−1^, *p* < 0.05) and that by the pith showed a similar but insignificant trend, whereas the whole straw and the reconstituted mixture exhibited lower NH_4_^+^ uptake (13.80 ± 0.23 → 11.70 mg·g^−1^ and 10.93 → 9.18 mg·g^−1^, respectively; Figure S4).

Such cross-effects may arise from changes in ionic strength, site occupation, counter-ion composition, or interfacial protonation. Among them, the uniform suppression of PO_4_^3−^ uptake is most plausibly attributed to the disruption of cation-bridging: PO_4_^3−^ is largely retained through divalent cations (Ca^2+^, Mg^2+^) associated with the biomass surface, and NH_4_^+^ can partially displace these cations via ion exchange, weakening the surface–cation–phosphate linkages; NH_4_^+^-HPO_4_^2−^ ion pairing in solution, which lowers the free phosphate activity, may further contribute. The enhanced NH_4_^+^ uptake by the isolated tissues is consistent with electrostatic co-adsorption, as the adsorbed phosphate species render the surface more negatively charged and provide additional binding sites for ammonium—an effect most pronounced for the mineral-rich rind—whereas for the whole straw this cooperative effect appears to be offset by the competition-related processes. The binary experiment therefore demonstrates matrix-dependent adsorption behavior rather than a specific competitive or cooperative mechanism; distinguishing among the possible contributions (e.g., by monitoring pH and Ca/Mg release during adsorption) requires further investigation.

### 3.4. Whole-Straw Adsorption Under Different Aqueous Conditions

**Figure 5:**
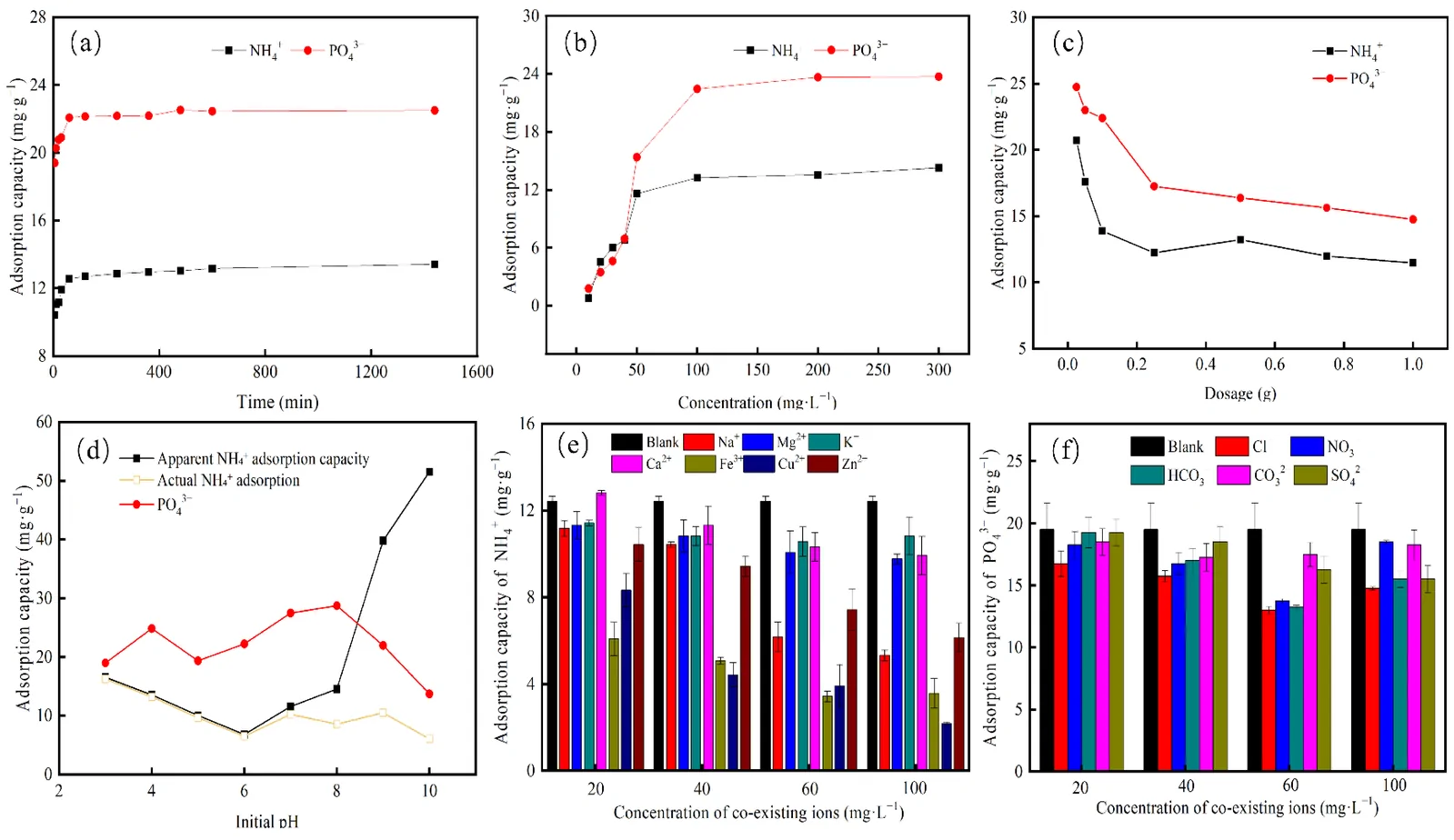
Adsorption behavior of whole Giant Juncao straw under different aqueous conditions. (**a**) Effect of contact time on NH_4_^+^ and PO_4_^3−^ uptake; (**b**) effect of initial solute concentration; (**c**) effect of adsorbent dosage; (**d**) effect of initial solution pH; (**e**) effect of coexisting cations on NH_4_^+^ uptake; (**f**) effect of coexisting anions on PO_4_^3−^ uptake.

After the tissue comparison, the remaining adsorption experiments focused on whole-straw powder. NH_4_^+^ and PO_4_^3^*^−^* uptake reached 12.55 and 20.07 mg·g^−1^, respectively, within the first 60 min, corresponding to approximately 93.5% and 90.0% of the respective 24 h values (13.42 and 22.30 mg·g^−1^) (Figure 5a). The rapid initial uptake is consistent with abundant readily accessible sites on external and fractured cell-wall surfaces, followed by slower redistribution among domains with different accessibility and affinity.

Increasing initial solute concentration to approximately 100 mg·L^−1^ produced a steep increase in uptake, after which the response approached a plateau (Figure 5b). At 300 mg·L^−1^, adsorption capacities were 14.27 mg·g^−1^ for NH_4_^+^ and 23.71 mg·g^−1^ for PO_4_^3−^. Within this range, the uptake curves approached but did not fully confirm a saturation plateau, which is acknowledged as a limitation of the present dataset. Nevertheless, the equilibrium uptake varied by less than 3% among the highest three initial concentrations, and the fitted saturation capacities (Section 3.6) were consistent with the largest directly observed uptakes, indicating that the fitted q_max_ values are anchored to the measured data rather than purely extrapolated. This behavior is consistent with progressive occupation of the accessible adsorption domain of the raw biomass under the tested conditions.

Increasing adsorbent dosage decreased adsorption capacity when expressed per unit mass (Figure 5c), a common consequence of increasing the ratio of available sites to solute and of incomplete utilization of the total solid surface at high dosage [32]. The dosage response is therefore most useful for defining an operating range and should not be interpreted by itself as evidence of a particular adsorption mechanism.

Initial pH affected ammonium and phosphate differently (Figure 5d). For NH_4_^+^, measured uptake decreased as pH increased to approximately 6 and then increased at higher pH. The rise at pH ≥ 8 requires particular caution: as the NH_4_^+^/NH_3_ equilibrium shifts toward NH_3_, volatilization can lower the measured aqueous ammoniacal-nitrogen concentration and thereby inflate an adsorption capacity calculated only from C_0_ − C_e_. However, the blank experiments without adsorbent revealed that a substantial fraction of NH_4_^+^ was lost via NH_3_ volatilization under alkaline conditions: the volatilization loss ratio was negligible at pH ≤ 6 (<5.0%) but increased sharply above pH 8, reaching 73.66% and 88.31% at pH 9 and 10, respectively (Figure S5). The high-pH increase therefore cannot be attributed unambiguously to adsorption. Phosphate uptake increased to a maximum measured value near pH 8 and then decreased. This trend is compatible with coupled changes in phosphate speciation (H_2_PO_4_^−^/HPO_4_^2−^), surface protonation, and mineral-mediated interactions rather than with a single electrostatic mechanism [33].

Coexisting ions also affected the two solutes differently (Figure 5e,f). For Na^+^, K^+^, Zn^2+^, Ca^2+^ and Mg^2+^, which remain fully dissolved at pH 6.0, the decreases in NH_4_^+^ adsorption shown in Figure 5e can be attributed primarily to competition for adsorption sites. For Cu^2+^ and particularly Fe^3+^, however, hydrolysis and (co)precipitation are thermodynamically possible at pH 6.0 at the applied concentrations (20–100 mg·L^−1^); therefore, the stronger inhibition observed for these two ions is interpreted conservatively as a combined effect of site competition and possible hydrolysis/(co)precipitation [34]. Common anions caused smaller overall changes in phosphate uptake, with clearer decreases at the higher co-ion concentrations. The persistence of measurable phosphate uptake in these matrices further indicates that the process cannot be reduced to a single nonspecific electrostatic interaction.

### 3.5. Adsorption Kinetics and Equilibrium Modeling

**Figure 6:**
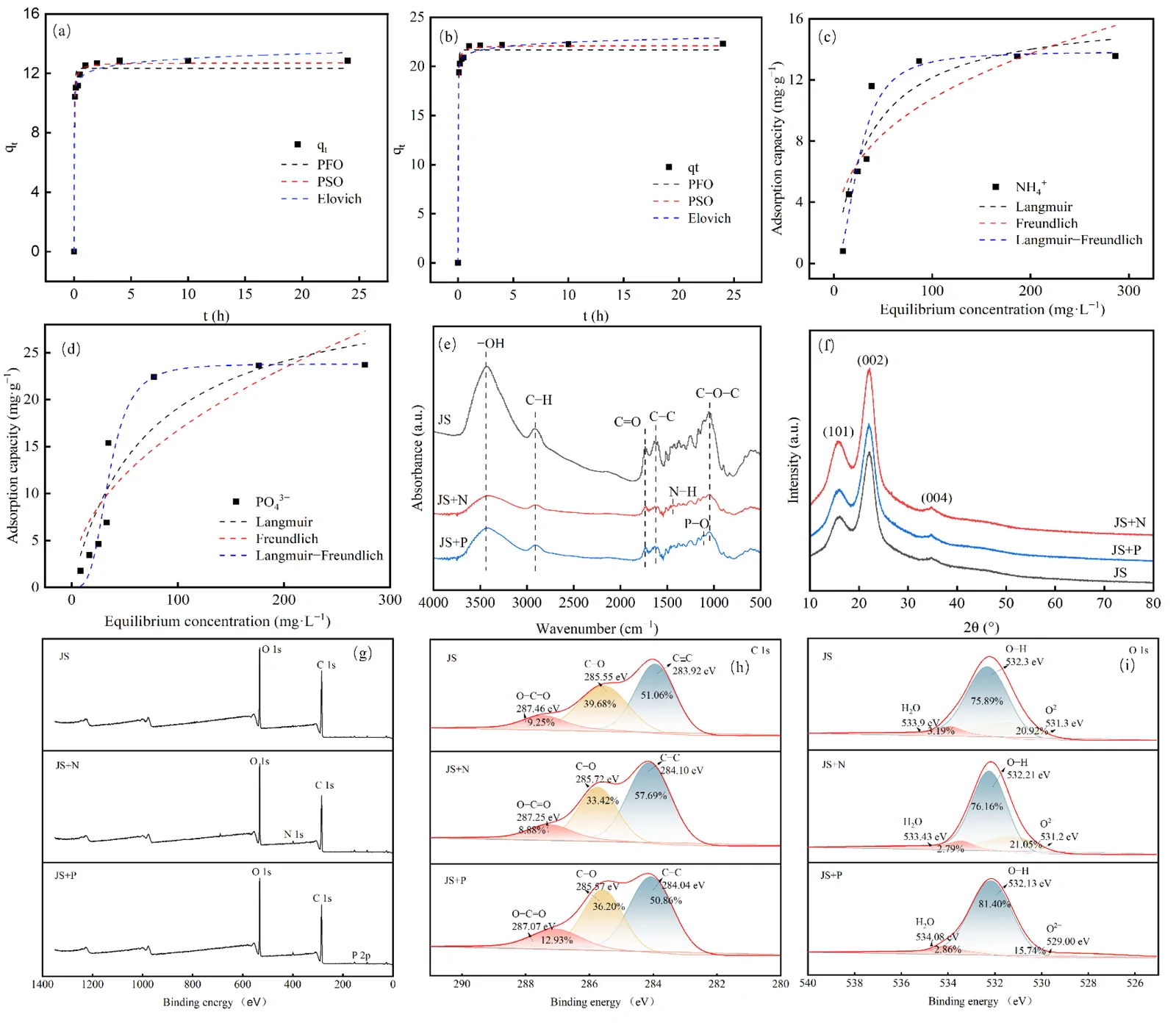
Kinetic and equilibrium modeling and spectroscopic changes associated with nutrient adsorption. (**a**,**b**) Pseudo-second-order linear representations for ammonium and phosphate, respectively; (**c**,**d**) equilibrium ammonium and phosphate data fitted with Langmuir, Freundlich, and Langmuir–Freundlich models; (**e**) FTIR spectra of Giant Juncao straw before and after ammonium or phosphate adsorption; (**f**) corresponding XRD patterns; (**g**) XPS survey spectra; (**h**) C 1s spectra; (**i**) O 1s spectra.

The kinetic data for both solutes were fitted by nonlinear regression directly to the original q_t_–t data. The PSO model provided the closest empirical description of the kinetic data for both solutes (Figure 6a,b), yielding the highest R^2^ (0.993 for NH_4_^+^ and 0.998 for PO_4_^3−^) and the lowest SSE, RMSE, and χ^2^ among the three models tested (Table S8). The calculated equilibrium capacities (12.71 and 22.11 mg·g^−1^ for NH_4_^+^ and PO_4_^3−^, respectively) were reasonably close to the experimental 24 h values (13.42 and 22.30 mg·g^−1^, Table S6), with the PSO estimates deviating by 5.3% and 0.9%, respectively, and being closer to the measured values than those of the PFO model. The Elovich model also described the uptake data well for both solutes (R^2^ = 0.991 for NH_4_^+^ and 0.996 for PO_4_^3−^; Table S7). The β values were similar for the two adsorbates (2.22 and 1.97 g·mg^−1^ for NH_4_^+^ and PO_4_^3−^, respectively), whereas α was very large in both systems (1.64 × 10^11^ and 9.04 × 10^17^ mg·g^−1^·min^−1^), indicating an extremely rapid initial uptake followed by a progressively slower rate as the available sites became occupied; such behaviour is typically associated with a energetically heterogeneous adsorbent surface. Importantly, the good PSO fit is treated here as a kinetic descriptor and not as independent evidence for chemisorption.

Equilibrium data were fitted with the Langmuir, Freundlich, and Langmuir–Freundlich equations (Figure 6c,d). In addition to R^2^, the isotherm models were compared using SSE, RMSE, and the Akaike Information Criterion (AIC), the last of which accounts for the number of fitted parameters (Tables S9 and S10). For both NH_4_^+^ and PO_4_^3−^, the Langmuir–Freundlich model yielded the lowest values of all three criteria (ΔAIC = 5.0 and 5.8 relative to the Langmuir model, respectively), and its predicted saturation capacities (13.86 and 23.80 mg·g^−1^ for ammonium and phosphate, respectively) agreed with the experimentally observed plateaus (13.57 and 23.71 mg·g^−1^) within 2% and 0.5%, whereas the Langmuir model overestimated qmax by ~22% and ~38%. Among the models tested, the combination of statistical and physical criteria therefore favors the Langmuir–Freundlich model as the best empirical description of the equilibrium adsorption of both adsorbates. Given the structural and chemical heterogeneity of raw giant juncao, the empirical flexibility of the three-parameter Langmuir–Freundlich equation is physically reasonable. However, differences in model fit alone do not establish monolayer adsorption, coordination bonding, cooperativity, or any other specific molecular mechanism. The fitted parameters are therefore used primarily as empirical descriptors of the measured equilibrium behavior of this heterogeneous biosorbent.

### 3.6. Spectroscopic Evidence and Plausible Adsorption Pathways

FTIR, XRD, and XPS provide converging evidence that ammonium adsorption is associated with oxygen-containing cell-wall environments, while stopping short of identifying a unique binding geometry. After ammonium adsorption, changes were observed in the broad O–H region and in the carbonyl/carboxylate region of the FTIR spectrum (Figure 6e). XPS showed an N 1s component near 400.7 eV consistent with ammonium-associated nitrogen, accompanied by changes in the relative C–O and O-containing components of the C 1s and O 1s spectra (Figure 6g–i). These observations are consistent with the retention of NH_4_^+^ in a protonated form in oxygen-rich (carboxyl/hydroxyl-rich) cell-wall environments, suggesting electrostatic association with negatively charged groups; however, the assignment of specific binding sites cannot be made from these spectra alone. However, FTIR and XPS alone cannot distinguish this pathway from ion exchange with endogenous counter-ions, which would produce essentially the same spectral changes; the relative contributions of the two mechanisms therefore cannot be separated on the basis of the present data, and their distinction would require, e.g., quantification of the concurrent release of endogenous base cations (K^+^, Ca^2+^, Mg^2+^, Na^+^). Hydrogen bonding may additionally contribute. XRD showed no new crystalline ammonium-containing phase (Figure 6f). The small change in apparent cellulose crystallinity (53.65% to 51.58%) is not, by itself, sufficient to demonstrate disruption of the crystalline hydrogen-bond network and is therefore not used as mechanistic proof.

For phosphate, the appearance of a P 2p signal near 133.2 eV after adsorption confirms surface-associated phosphate, while FTIR changes in the O–H and 1000–1100 cm^−1^ regions are consistent with altered oxygen-containing environments (Figure 6e,g). Changes in the C 1s and O 1s component distributions further support participation of surface oxygen functionalities. However, these spectral changes cannot by themselves distinguish hydrogen bonding from ligand exchange or cation bridging. No crystalline calcium-, magnesium-, or ammonium-phosphate phase was detected by XRD, so precipitation of an amorphous phosphate phase cannot be established from the present data. The endogenous Ca^2+^/Mg^2+^ in the straw tissues may contribute to phosphate retention via electrostatic attraction, inner-sphere complexation, or formation of Ca-P/Mg-P precipitates, as reported for related biomass-derived sorbents [25,35]. Together with the negative zeta potential, the data support a heterogeneous, non-single-mechanism picture in which direct nonspecific anion attraction is unlikely to be the sole pathway. In summary, the FTIR, XPS, and XRD results provide converging but not mechanistically unique evidence: ion exchange, electrostatic attraction, hydrogen bonding, ligand exchange, and amorphous precipitation may produce similar spectral responses, and these techniques alone cannot distinguish among them. The mechanistic assignments above should therefore be regarded as plausible interpretations supported jointly by the batch adsorption data and the spectral evidence; unambiguous discrimination (e.g., quantitative analysis of concurrent counter-ion release and its stoichiometric relationship with the uptake, or comparison before and after selective blocking of individual functional groups) merits further investigation.

It should be emphasized that the present results were obtained in well-defined synthetic NH_4_^+^/PO_4_^3−^ solutions. Real wastewaters contain competing ions, dissolved organic matter, and suspended solids, and exhibit fluctuating pH; these factors may reduce the uptake capacity and alter selectivity, so the reported capacities should be regarded as reference values for single-solute conditions rather than predictions of field performance. The present study focuses on the structural heterogeneity between the rind and pith of Giant Juncao straw, their comparative NH_4_^+^ and PO_4_^3−^ uptake performance, and the underlying mechanisms, thereby providing a basis for subsequent modification of the straw to further enhance its adsorption capacity. For practical nutrient recovery, the nutrient-loaded straw could be directly returned to soil or composted as a slow-release nutrient carrier; alternatively, pyrolytic conversion into biochar would allow the retained N and P to be further enriched and stabilized for field application. Modification-based performance enhancement and field evaluation of the nutrient-loaded straw are the subject of our ongoing work.

## 4. Conclusions

Giant Juncao straw is a strongly heterogeneous lignocellulosic feedstock rather than a compositionally uniform adsorbent. The pith occupies most of the stem volume but contributes relatively little dry mass and is characterized by thin-walled parenchyma, very low density, greater N_2_-accessible surface/pore volume, higher pectin content, lower lignification, and lower cellulose crystallinity than the rind. These tissue differences were reflected most clearly in ammonium uptake: isolated pith adsorbed substantially more NH_4_^+^ than rind, whereas phosphate adsorption differed only modestly between the two tissues. Whole-straw NH_4_^+^ uptake exceeded both isolated fractions and the simple mass-weighted expectation, producing a non-additive response that is potentially useful but requires independent validation before being assigned a synergistic mechanism. More than 90% of the 24 h uptake occurred within 60 min, and PSO kinetics and Langmuir–Freundlich isotherms described the measured data well as empirical models. FTIR and XPS changes support involvement of oxygen-containing cell-wall environments in nutrient retention, but the present data do not uniquely establish outer-sphere/inner-sphere complexation, ligand exchange, or amorphous precipitation. Overall, the study shows that native tissue architecture and lignocellulosic chemistry jointly shape nutrient adsorption by minimally processed Giant Juncao. Future work should prioritize real-wastewater validation, mass-balance controls for ammonium at alkaline pH, adsorption–desorption/reuse tests, and evaluation of nutrient-loaded biomass for safe resource recovery.

## Data Availability

The data supporting the findings of this study are available from the corresponding authors upon reasonable request.

## Supplementary Materials

The following supporting information can be downloaded at: https://www.mdpi.com/article/10.3390/polym18202594/s1, The Supporting Information includes supplementary kinetic and isotherm equations. Figure S1. Detailed XRD peak fitting of cellulose in Giant Juncao rind and pith. Diffraction components assigned in the source dataset to the (1–10), (110), (200), and (004) planes are shown for (a) rind and (b) pith. Figure S2. Side-chain regions of 2D-HSQC NMR spectra of Giant Juncao (a) rind and (b) pith. Figure S3. Release of NH^4+^ and PO_4_^3−^ by water extraction from whole stem, rind, and pith of giant juncao. Figure S4. NH^4+^ and PO_4_^3−^ adsorption capacities of the physical mixture (Mix-JS, rind:pith = 0.7315:0.2685) in single-solute and binary systems. Figure S5. The adsorption capacity of NH^4+^ (a) and NH_3_ volatilization loss (b) under different pH conditions. Table S1. Physical properties of Giant Juncao rind and pith. Table S2. Temperature ranges and mass loss during thermogravimetric analysis of Giant Juncao rind and pith. Table S3. Pyrolysis-characteristic parameters of Giant Juncao rind and pith at β = 10 °C min^−1^. Table S4. Apparent cellulose crystallite dimensions and interplanar spacings from XRD peak fitting. Table S5. Semiquantitative lignin interunit-linkage and aromatic-unit distributions derived from 2D-HSQC NMR. Table S6. Fitting parameters for NH^4+^ and PO_4_^3−^ kinetic models. Table S7. Parameters of the Elovich model. Table S8. Goodness-of-fit and error function analysis for the kinetic models fitted to the NH^4+^ and PO_4_^3−^ adsorption kinetics onto Giant Juncao straw. Table S9. Isotherm-fitting parameters for ammonium and phosphate adsorption by raw Giant Juncao straw. Table S10. Parameters and goodness-of-fit for the isotherm models fitted to the equilibrium data of NH^4+^ and Phosphate by nonlinear regression.
