# Supplementary material for: Tissue-Level Heterogeneity in Raw Giant Juncao Straw and Its Influence on Ammonium and Phosphate Adsorption
Source: Polymers (Basel). 2026 Oct 21;18(20):2594. doi: 10.3390/polym18202594 (PMC13708977; doi:10.3390/polym18202594)
Supplement: Supplementary file 1 [file polymers-18-02594-s001.zip › polymers-4624333-supplementary.pdf]

Supporting Information

Tissue-Level Heterogeneity in Raw Giant Juncao Straw and Its Influence on Ammonium and Phosphate Adsorption

Xiaomei Wu[1,3], Chaohui Chen[1,2], Wenting Ren[1,2] , Rilong Yang[1,2], Junyue Shan[1,2,*], Yan Yu[1,2,*]

1 College of Materials Engineering, Fujian Agriculture and Forestry University, Fuzhou 350108, China
2 Key Laboratory of Plant Fiber Functional Materials, National Forestry and Grassland Administration, Fuzhou 350002, China
3 Institute of Food Science and Technology, Fujian Academy of Agricultural Sciences, Fuzhou 350003, China
* Correspondence: Sjy20251017@163.com (J.S.);yuyan9812@outlook.com (Y.Y.)

Contents

• Figures S1–S5
• Tables S1–S10

Supplementary Methods

S1. Kinetic and Equilibrium Models

The uptake data were fitted using the pseudo-first-order (PFO), pseudo-second-order (PSO), and Elovich models used in the source dataset:

$$q_t = q_e(1 - e^{-k_1 t}) \quad \text{(S1)}$$

$$\frac{t}{q_t} = \frac{1}{k_2 q_e^{\,2}} + \frac{t}{q_e} \quad \text{(S2)}$$

$$q_t = (1/\beta)ln(\alpha\beta) + (1/\beta)lnt \quad \text{(S3)}$$

Here, k_1 and k_2 are the PFO and PSO rate constants, respectively; α ($mg{\cdot}g^{-1}{\cdot}h^{-1}$) represents the initial adsorption rate. β ($g{\cdot}mg^{-1}$) is related to the extent of surface coverage and the activation energy of chemisorption; a larger β indicates a more rapid decrease in adsorption rate with increasing surface coverage.Model fitting is used descriptively; a high PSO R^2 is not treated as independent proof of chemisorption.

Equilibrium data were fitted with Langmuir, Freundlich, and Langmuir–Freundlich models. The functional forms used for the fits are:

$$\frac{C_e}{q_e} = \frac{1}{q_{max} K_L} + \frac{C_e}{q_{max}} \quad \text{(S4)}$$

$$q_e = K_F C_e^{1/n} \quad \text{(S5)}$$

$$q_e = \frac{q_{max} K_{LF} C_e^{\,n}}{1 + K_{LF} C_e^{\,n}} \quad \text{(S6)}$$

K_L, K_F, and K_{LF} are fitted adsorption constants and qmax is the fitted capacity parameter. The Langmuir–Freundlich exponent n is treated as an empirical parameter describing the flexibility of the heterogeneous equilibrium response. Because raw Giant Juncao contains multiple tissues and chemical domains, the fitted models are interpreted as phenomenological descriptors rather than unique molecular mechanisms.

Supplementary Figures

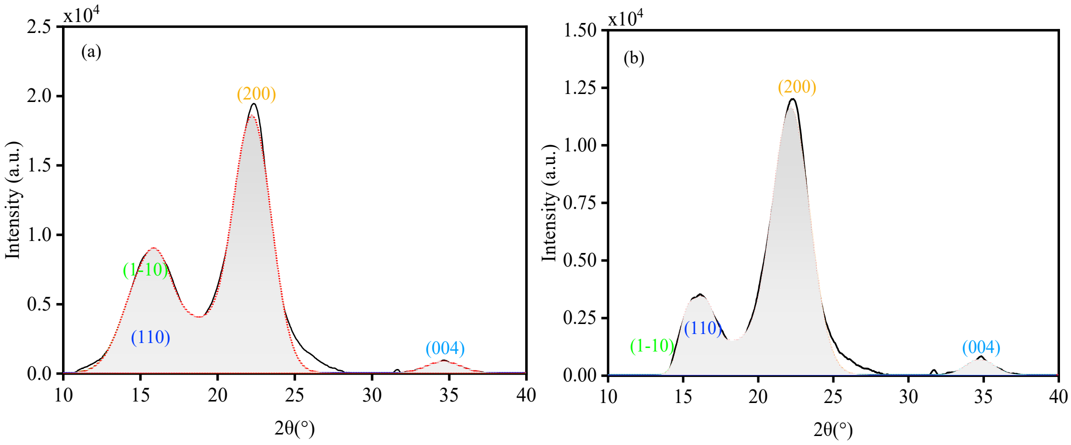

Figure S1. Detailed XRD peak fitting of cellulose in Giant Juncao rind and pith. Diffraction components assigned in the source dataset to the (1–10), (110), (200), and (004) planes are shown for (a) rind and (b) pith. The corresponding apparent crystallite dimensions and interplanar spacings are summarized in Table S4.

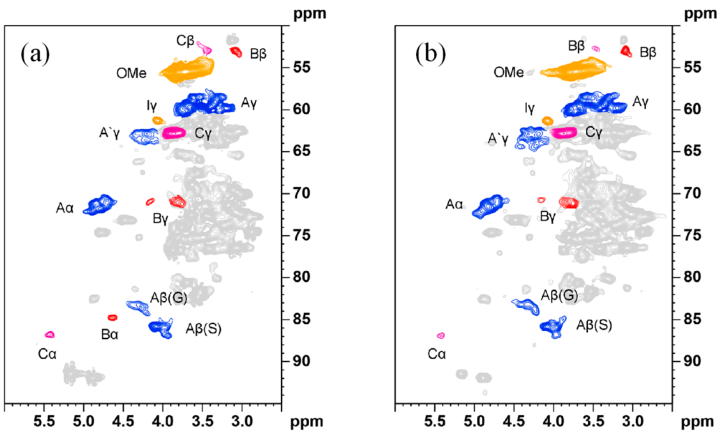

Figure S2. Side-chain regions of 2D-HSQC NMR spectra of Giant Juncao (a) rind and (b) pith.

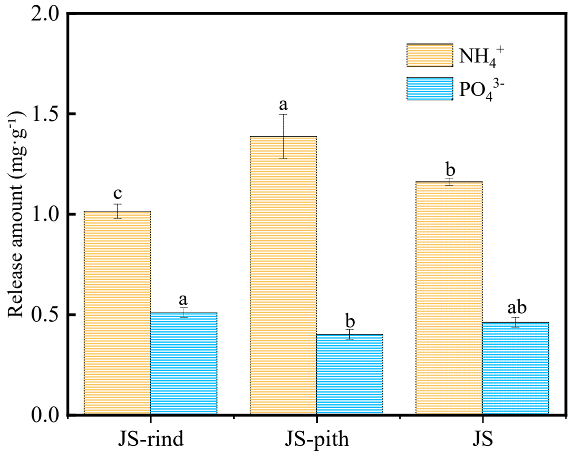

Figure S3. Release of NH_4^+ and PO_4^{3-} by water extraction from whole stem, rind, and pith of giant juncao. Bars with the same letter are not significantly different ($p > 0.05$); bars with different letters are significantly different ($p< 0.05$).

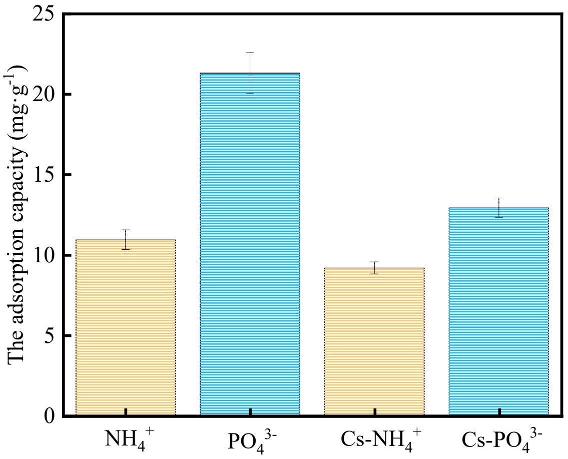

Figure S4. NH_4^+ and PO_4^{3-} adsorption capacities of the physical mixture (Mix-JS, rind : pith = 0.7315 : 0.2685) in single-solute and binary systems. Error bars represent standard deviations (n = 3).

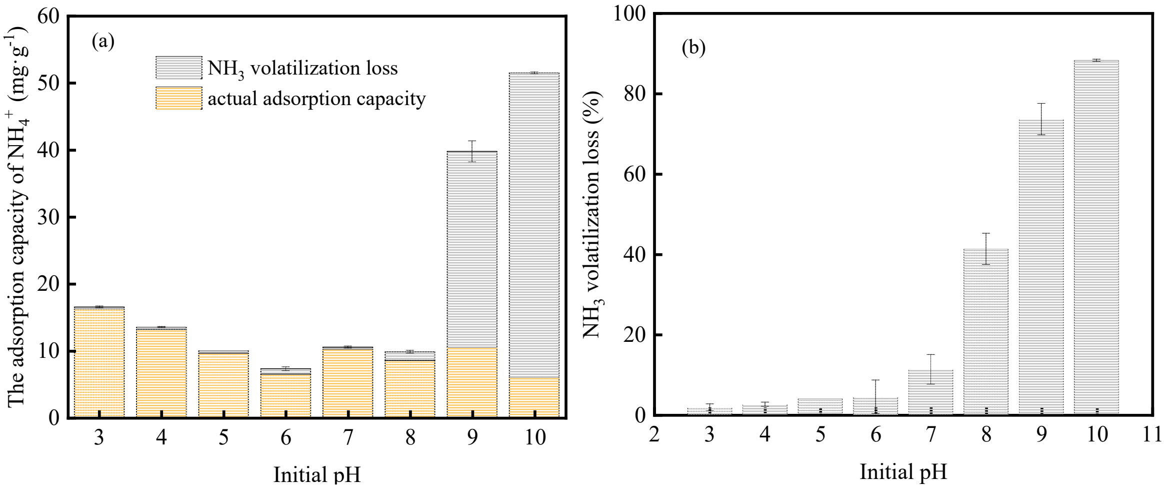

Figure S5. The adsorption capacity of NH_4^+(a) and NH_3 volatilization loss(b) under different pH conditions

Supplementary Tables

Table S1. Physical properties of Giant Juncao rind and pith.

Tissue	Moisture content (%)	Basic density ($g\ cm^{-3}$)	pH	Mass ratio (%)	Volume ratio (%)
Rind	45.15 ± 4.19	0.570 ± 0.011	6.3	73.15	27.67
Pith	41.23 ± 4.21	0.072 ± 0.0068	6.7	26.85	72.33

Table S2. Temperature ranges and mass loss during thermogravimetric analysis of Giant Juncao rind and pith.

Tissue	Drying range (°C)	Loss (%)	Transition range (°C)	Loss (%)	Main pyrolysis range (°C)	Loss (%)	High-T range (°C)	Loss (%)	Total loss (%)
Rind	25–120	5.27	120–190	0.65	190–420	66.62	420–901	13.22	85.75
Pith	25–112	5.73	112–185	0.21	185–411	70.84	411–901	12.35	89.13

Table S3. Pyrolysis-characteristic parameters of Giant Juncao rind and pith at β = 10 °C min^{-1}.

Tissue	T (°C)	Tmax (°C)	(dw/dt)max (% min^{-1})	V∞	(dw/dt)mean (% min^{-1})	ΔT1/2 (°C)	D
Rind	190	351	−7.77	46.81	−0.97	311	1.70×10^{-5}
Pith	185	346	−10.18	46.70	−1.01	308	2.42×10^{-5}

Table S4. Apparent cellulose crystallite dimensions and interplanar spacings from XRD peak fitting.

Crystal plane	D, rind (Å)	D, pith (Å)	d, rind (Å)	d, pith (Å)
(1–10)	24.21	62.04	5.79	5.83
(110)	39.90	45.77	5.44	5.40
(200)	27.70	26.99	3.99	4.00
(004)	31.98	37.41	2.59	2.58

Table S5. Semiquantitative lignin interunit-linkage and aromatic-unit distributions derived from 2D-HSQC NMR.

Tissue	A (β-O-4′)	B (β-β′)	C (β-5′)	S-type	G-type	H-type	S/G
Rind	49.94 (79.15%)	7.35 (11.65%)	5.81 (9.20%)	46.46%	48.54%	5.00%	0.96
Pith	51.68 (92.49%)	ND	4.19 (7.51%)	40.81%	53.21%	5.98%	0.77

Table S6. Fitting parameters for NH_4^+ and PO_4^{3-} kinetic models.

Adsorbate	q_e,exp ($mg \cdot g^{-1}$)	PFO k_1	PFO q_e,cal ($mg \cdot g^{-1}$)	PFO R^2	PSO k_2	PSO q_e,cal ($mg\ g^{-1}$)	PSO R^2
NH_4^+	13.42	20.36 ± 3.51	12.34 ± 0.24	0.9756	3.53 ± 0.59	12.71 ± 0.15	0.9933
PO_4^{3-}	22.30	21.68 ± 0.26	0.84	0.9903	3.38 ± 0.48	22.11 ± 0.15	0.9977

Table S7. Parameters of the Elovich model.

Adsorbate	α<br>$mg \cdot g^{-1} \cdot h^{-1}$	β<br>$g \cdot mg^{-1}$	R^2
NH_4^+	$1.65\times10^{11} \pm 7.03\times10^{11}$	2.22 ± 0.37	0.9909
PO_4^{3-}	9.04×10^{17}	1.97 ± 0.34	0.9959

Table S8 Goodness-of-fit and error function analysis for the kinetic models fitted to the NH_4^+ and PO_4^{3-} adsorption kinetics onto Giant Juncao straw (n = 10, nonlinear regression)

Model	NH_4^+				PO_4^{3-}			
	R^2	**SSE**	**RMSE ($mg \cdot g^{-1}$)**	AIC	R^2	**SSE**	**RMSE ($mg \cdot g^{-1}$)**	AIC
PFO	0.9756	3.36	0.579	0.268	0.9903	4.048	0.636	0.185
PSO	0.9933	0.95	0.309	0.076	0.9977	0.982	0.313	0.045
Elovich	0.9909	1.26	0.355	0.101	0.9959	1.708	0.413	0.078

Table S9. Isotherm-fitting parameters for ammonium and phosphate adsorption by raw Giant Juncao straw.

Adsorbate	Langmuir		Freundlich		Langmuir-Freundlich		
	**K_L ($L\ mg^{-1}$)**	**q_{max} ($mg\ g^{-1}$)**	**K_F**	**1/n**	**q_{max} ($mg\ g^{-1}$)**	**K_{LF} $(L\ mg^{-1})^n$**	**n**
NH_4^+	0.028	16.52	2.16	0.35	13.86	8.18×10^{-4}	2.19
PO_4^{3-}	0.014	32.62	1.82	0.48	23.80	3.28×10^{-6}	3.54

Table S10. Parameters and goodness-of-fit for the isotherm models fitted to the equilibrium data of NH_4^+ and Phosphate by nonlinear regression (n = 8 per adsorbate)

Model	NH_4^+				PO_4^{3-}			
	R^2	**SSE**	**RMSE ($mg \cdot g^{-1}$)**	AIC	R^2	**SSE**	**RMSE ($mg \cdot g^{-1}$)**	AIC
Langmuir	0.8712	21.48	1.64	11.90	0.8549	93.80	3.42	23.69
Freundlich	0.7313	44.80	2.37	17.78	0.7675	150.28	4.33	27.46
Langmuir–Freundlich	0.9466	8.90	1.06	6.86	0.9450	35.56	2.11	17.93
